# An evaluation of the potential factors affecting lifetime reproductive success in salmonids

**DOI:** 10.1111/eva.13263

**Published:** 2021-06-29

**Authors:** Ilana J. Koch, Shawn R. Narum

**Affiliations:** ^1^ Columbia River Inter‐Tribal Fish Commission Hagerman ID USA

**Keywords:** fitness, migration, origin, review, salmon, size

## Abstract

Lifetime reproductive success (LRS), the number of offspring produced over an organism's lifetime, is a fundamental component of Darwinian fitness. For taxa such as salmonids with multiple species of conservation concern, understanding the factors affecting LRS is critical for the development and implementation of successful conservation management practices. Here, we reviewed the published literature to synthesize factors affecting LRS in salmonids including significant effects of hatchery rearing, life history, and phenotypic variation, and behavioral and spawning interactions. Additionally, we found that LRS is affected by competitive behavior on the spawning grounds, genetic compatibility, local adaptation, and hybridization. Our review of existing literature revealed limitations of LRS studies, and we emphasize the following areas that warrant further attention in future research: (1) expanding the range of studies assessing LRS across different life‐history strategies, specifically accounting for distinct reproductive and migratory phenotypes; (2) broadening the variety of species represented in salmonid fitness studies; (3) constructing multigenerational pedigrees to track long‐term fitness effects; (4) conducting LRS studies that investigate the effects of aquatic stressors, such as anthropogenic effects, pathogens, environmental factors in both freshwater and marine environments, and assessing overall body condition, and (5) utilizing appropriate statistical approaches to determine the factors that explain the greatest variation in fitness and providing information regarding biological significance, power limitations, and potential sources of error in salmonid parentage studies. Overall, this review emphasizes that studies of LRS have profoundly advanced scientific understanding of salmonid fitness, but substantial challenges need to be overcome to assist with long‐term recovery of these keystone species in aquatic ecosystems.

## 1 INTRODUCTION

Lifetime reproductive success (LRS) is broadly defined as the number of offspring an individual produces over the span of a lifetime (Clutton‐Brock, 1988) and is generally considered a sufficient estimate of fitness in natural populations (Arnold & Wade, 1984; Grafen, 1988). LRS is thought to consist of two main components which intrinsically connect both sexual and natural selection. The first component is the number of offspring produced, with the caveat that distinct evolutionary trade‐offs exist between offspring number and quality, and the second component is the number of available reproductive seasons within an individual's lifetime (Barrowclough & Rockwell, 1993; Olofsson et al., 2009; Philippi & Seger, 1989). In order to have evolutionary consequences, individual phenotypic differences in LRS need to be heritable across generations (Shuster & Wade, 2003).

Although LRS is a commonly used estimation of fitness across taxonomic groups, gathering LRS data has presented a challenge in natural populations, particularly for species with external fertilization and no postnatal parental care. A direct count of the number of offspring requires both confidence in sampling and accuracy in parentage assignment once sampled. Furthermore, following an individual throughout their lifespan can be technically challenging, costly, and in many cases, simply not possible. Therefore, constraints on technical sampling of parents in natural environments have led some studies to rely on a correlate or “proxy” measure of fitness, such as morphology, phenology, performance measures, and various life‐history traits (some examples would include body mass, seasonal timing, growth rate, and offspring size; Barrowclough & Rockwell, 1993; Franklin & Morrissey, 2017; Kingsolver et al., 2012).

Advances in genetic sequencing technology have provided a cost‐effective approach to determining the pedigree structure of individuals within a population (DeWoody, 2005; Johnson et al., 2019; Jones et al., 2010). As the financial burden of genetic sampling and subsequent parentage assignment has decreased over the last 20 years, there have been a number of studies providing direct estimates of LRS, ranging from insects (Ingram et al., 2013; Rodríguez‐Muñoz et al., 2010), birds (Costanzo et al., 2017; Eastwood et al., 2019; Gienapp & Merilä, 2011; Pärn et al., 2009; Schroeder et al., 2015; Webster et al., 2007), and reptiles (Le Galliard et al., 2008; Warner & Shine, 2008), to mammals, both small (Dugdale et al., 2010, 2011; Lardy et al., 2015; Marshall et al., 2017; Schradin & Lindholm, 2011) and large (Lancaster et al., 2007; Markussen et al., 2019; Setchell et al., 2005; Sparkman et al., 2010; Spiering et al., 2011).

Salmonids represent an ideal study system to estimate pedigree structure, reliably measure LRS, and address evolutionary questions given their unique life‐history strategies and population structure, high fecundity, wide variability in life‐history traits, and the wealth of genetic information gained from commercial and conservation aquaculture (Stearns & Hendry, 2004). Additionally, salmonids demonstrate very high individual‐ and population‐level variability in LRS, intense intrasexual competition, and high mortality overall, making them also ideal for addressing questions in sexual selection (Fleming & Reynolds, 2004).

Variation in numerous life‐history traits occurs both within and across salmonid species, including both semelparous and iteroparous reproductive strategies (Quinn, 2005; see Box 1 for glossary of terms presented). Semelparity is characterized by a single reproductive event, followed by death, and represents a life‐history strategy that can be found across the taxonomic spectrum (Braithwaite & Lee, 1979; Crespi & Teo, 2002; Fritz et al., 1982; Young & Augspurger, 1991). Multiple species of salmonid fishes exhibit a semelparous life history, but it is particularly common in the genus *Oncorhynchus*, including Chinook (*Oncorhynchus tshawytscha*), Coho (*Oncorhynchus kisutch*), Sockeye (*Oncorhynchus nerka*), Pink (*Oncorhynchus gorbuscha*), and Chum (*Oncorhynchus keta*) Salmon (Crespi & Teo, 2002). Since semelparous species of salmonids reproduce offspring during a single reproductive bout prior to death, LRS can be estimated by evaluating a single breeding season. Alternatively, salmonids exhibiting the potential for iteroparity are characterized by repeat breeding episodes, the number of which depends on numerous factors including sex, size, and anthropogenic impacts (Fleming, 1998). Some examples of iteroparous salmonids include European Grayling (*Thymallus thymallus*), Lake Whitefish (*Coregonus clupeaformis*), Rainbow and Steelhead Trout (*Oncorhynchus* *mykiss*), Cutthroat Trout (*Oncorhynchus* *clarkii*), Lake Charr (*Salvelinus namaycush*), Arctic Charr (*Salvelinus alpinus*), Brook Charr (*Salvelinus fontinalis*), Bull Trout (*Salvelinus confluentus*), and Dolly Varden Charr (*Salvelinus malma*), Atlantic Salmon (*Salmo salar*), and Brown Trout (*Salmo trutta*) (Crespi & Teo, 2002; Johnston et al., 2007; Lambert & Dodson, 1990; Northcote, 1995) among others. Because it is more challenging to estimate LRS for iteroparous species which require more than one sampling effort over the lifetime of the fish, only a limited number of studies have accounted for multiple spawning events (Christie et al., 2018; Seamons & Quinn, 2010). Numerous hypotheses have been proposed regarding the evolution of semelparity versus iteroparity in salmonids (Crespi & Teo, 2002; Hutchings & Morris, 1985; Schaffer, 2004) and with each strategy demonstrating distinct trade‐offs, such as those involved in energy expenditure (Fleming, 1998; Fleming & Reynolds, 2004). Within iteroparous salmonids, trade‐offs exist between survival and reproduction later in life (Christie et al., 2018; Seamons & Quinn, 2010).

BOX 1 **Glossary of fisheries terms listed in alphabetical order**

**Anadromous**

Fish born in freshwater, followed by growth and rearing in saltwater, and a return to freshwater to reproduce.

**Broodstock**

Group of reproductively mature fish used for artificial spawning in the hatchery setting; parents of hatchery‐origin fish.

**Hatchery‐origin**

Fish that were born in the hatchery through artificial spawning.

**Integrated Broodstock**

Hatchery population created by varying proportions of hatchery‐origin and natural‐origin fish. Fully integrated hatchery programs use only natural‐origin fish in broodstock. Typically observed in supplementation programs.

**Iteroparity**

A life‐history strategy characterized by multiple reproductive events across more than one spawning season.

**Lifetime Reproductive Success (LRS)**

As defined in this study, the total number of offspring produced over the adult lifetime.

**Local Hatchery Stock**

Broodstock created from naturally spawning fish inhabiting streams that are the same as release location of artificially spawned juveniles.

**Natural‐Origin**

As defined in this study, fish that were born in the natural environment regardless of the origin of parents or grandparents. Note: some studies reviewed here refer to fish as “wild” despite unknown origin of parents or grandparents, but we use “natural‐origin” since “wild” implies lack of any ancestry from hatchery‐origin fish which is typically unknown.

**Nonlocal Hatchery Stock**

Broodstock created from fish inhabiting streams that are different from release location of artificially spawned juveniles.

**Relative Reproductive Success (RRS)**

The average number of offspring produced by hatchery‐origin fish compared to the average number of offspring produced by natural‐origin fish. Can be calculated as relative LRS. Also referred to as “relative fitness”.

**Resident Fish**

Fish that do not migrate to the ocean but remain in freshwater to live and spawn.

**Segregated Broodstock**

Hatchery population created solely by crosses of hatchery‐origin fish as opposed to natural‐origin. Typically observed in traditional hatchery programs.

**Semelparity**

A life‐history strategy characterized by death following spawning in a single reproductive season.

Salmonids also display a vast array of other alternative life‐history strategies that can impact LRS, such as alternative reproductive phenotypes and alternative migratory tactics. For example, precocial males generally mature (i.e., produce milt) at least a year earlier than average adult males and, depending on the species, can either migrate to the ocean and return early (commonly referred to as “jack” males), partially migrate (“minijacks”), or mature entirely in freshwater (“residents”) (Johnson et al., 2012; Mullan et al., 1992; Pearsons et al., 2009; Zimmerman et al., 2003). Precocial maturation has often been used to describe resident males that produce milt as parr, but in this review we use the term “precocial” more generally to refer to males that mature at least a year earlier than average adult males of a given species. This precocial maturation, or “jacking,” is in contrast to larger and older “hooknose” males observed in semelparous Pacific Salmon *Oncorhynchus* spp. (Allen et al., 2007; Gross, 1985; Quinn & Foote, 1994) and other anadromous salmonids, such as Atlantic Salmon. For example, in Atlantic Salmon, both size and age at maturity can vary widely, with some males maturing as precocial parr in their natal streams and using a sneaker strategy to fertilize eggs compared to a fighter strategy displayed in larger anadromous males (Fleming, 1996; Fleming & Reynolds, 2004). Precocial maturation also occurs in other iteroparous salmonid species, such as Steelhead Trout (Viola & Schuck, 1995; Willson, 1997). Similar to males, there are a few salmonid species that display precocial maturation of females as well and are commonly referred to as “jills” (Willson, 1997). Previous work has suggested that alternative reproductive phenotypes in salmonids are heritable and maintained through the processes of both negative frequency‐dependent selection and condition‐dependent sexual selection (Berejikian et al., 2010, 2011; Christie et al., 2018; DeFilippo et al., 2019; Fleming, 1996; Gross, 1985, 1996; Heath et al., 2002; Reed et al., 2019; Taborsky, 2008; Tentelier et al., 2016). One proposed evolutionary explanation for the presence of mature male parr on the spawning grounds is that they can increase overall genetic diversity and reduce inbreeding since they are unlikely to mate with females from their own cohort (i.e., full‐ or half‐siblings; Perrier et al., 2014).

Alternative migratory tactics also include other forms of life‐history variation within salmonids that, similar to alternative reproductive behavior, are determined by both environmental influences and genetically based developmental thresholds (Dodson et al., 2013; Kendall et al., 2015) and can occur along a continuum both within and across populations (Jonsson & Jonsson, 1993). Anadromous salmonids spend part of their life in freshwater (postnatal development and spawning) and the other part in the ocean where they develop and grow, while resident fish spend the entirety of their lifecycle in freshwater. While the majority of species within the genus *Oncorhynchus* display anadromy (ex. Chinook, Chum, Coho, and Pink Salmon), other species such as Coastal Cutthroat Trout, Steelhead Trout, and Sockeye Salmon display both an anadromous and a resident form (Dodson et al., 2013; Pavlov & Savvaitova, 2008; Quinn & Myers, 2004; Trotter, 1989). Similarly, *Salmo*, *Salvelinus*, *Thymallus*, and *Coregonus* species display a continuum of anadromy and residency (Brenkman & Corbett, 2005; Dodson et al., 2013; Jonsson et al., 1988; Klemetsen et al., 2003; Larsson et al., 2013; Morin et al., 1982; Northcote, 1995; Pavlov & Savvaitova, 2008).

Multiple species of salmonids, including Atlantic Salmon, Steelhead Trout, Chinook Salmon, Chum Salmon, Coho Salmon, and Sockeye Salmon, are considered of high concern for conservation (International Union for Conservation of Nature, IUCN; U.S. Endangered Species Act, ESA; Canadian Species at Risk Act, SARA) owing to numerous anthropogenic impacts such as dam construction, overfishing, climate change, and habitat degradation (Boisclair, 2004; Gustafson et al., 2007). Estimating productivity of conservation species provides a vital component of population viability (Garcia de Leaniz et al., 2007; Waples & Hendry, 2008), with the success of conservation management frequently measured by LRS. Therefore, understanding the vast array of recent literature that has addressed salmonid reproductive ecology can provide conservation managers with a framework for constructing successful management plans and assessing success of existing conservation strategies.

Here, we synthesize peer‐reviewed studies from the published literature over the last two decades (since the year 2000) that assessed the factors affecting adult‐to‐adult LRS (i.e., fitness) across salmonids that display a range of life‐history strategies using parentage assignment from adult fish to their adult offspring with a few important caveats. First, our primary focus is on species from the genus *Oncorhynchus*, as the LRS literature is heavily biased toward these species, particularly due to their conservation status. However, we also provide studies that estimated LRS in the genus *Salmo* (see Box 2 for a glossary of the primary salmonid species presented in this review). Second, the majority of the studies examined in this review estimated LRS during the span of one breeding season for both semelparous and iteroparous species. However, while most LRS studies in iteroparous species did not account for repeat spawning, the occurrence of repeat spawning is quite low for anadromous salmonids (generally <10% in Steelhead Trout populations; Busby et al., 1996; Christie et al., 2018; Seamons & Quinn, 2010). Therefore, we provide the caveat that although an estimate of offspring number is a true measure of LRS in semelparous species, it may not necessarily accurately reflect LRS in iteroparous species which can spawn in more than one breeding season. Third, while the ultimate goal in salmonid fitness studies is to estimate adult‐to‐adult LRS for multiple generations, sampling juvenile offspring offers a more tractable alternative for studies that do not have capture locations to sample returning adult anadromous fish. Additionally, adult‐to‐juvenile LRS estimates can provide larger sample sizes in LRS studies because juveniles are sampled prior to outmigration and therefore are largely absolved from extraneous factors that could impact returning adult sampling and survival, such as straying or prespawn mortality (Keefer & Caudill, 2014). While there have been very few studies that have directly compared the factors affecting both adult‐to‐juvenile and adult‐to‐adult LRS within the same study system (Berntson et al., 2011; Ford et al., 2006, 2012; Kostow et al., 2003), results suggest that adult‐to‐juvenile estimates provide comparable results and can be extremely informative for study systems that do not have access to adult‐to‐adult data (Berntson et al., 2011). Therefore, in addition to adult offspring produced from adult parents (i.e., adult‐to‐adult; Table 1), we also include results of juvenile offspring produced from adult parents (i.e., adult‐to‐juvenile; Table 2).

**TABLE 1:** Summary of studies estimating lifetime reproductive success (adult‐to‐adult) across species

Species	Reference	General findings
Atlantic Salmon (*Salmo salar*)	McGinnity et al. (2003)	Experimental crosses were made between farmed and wild fish. All crosses containing farmed fish (with the exception of backcross to wild) across one and two generations demonstrated significantly lower LRS compared to wild fish
	McGinnity et al. (2004)	LRS was compared between wild, ranched, and non‐native fish. No differences in LRS between native and ranched. The non‐native group demonstrated significantly lower LRS than both the native and ranched group
	O’Sullivan et al. (2020)	Significantly lower LRS for captive‐bred compared to wild‐bred fish in five out of six spawning cohorts with males and females combined. When sexes were separated, female captive‐bred LRS was significantly lower in four spawning cohorts and in two spawning cohorts for males. Annual population productivity was lower in years where captive‐bred fish represented a greater proportion of potential spawners
Chinook Salmon (*Oncorhynchus tshawytscha*)	Ford et al. (2012)	Negative relationship between the LRS of individuals bred in captivity and the LRS of their male offspring in the wild. Did not find same result for female offspring. Includes additional estimates of adult‐to‐juvenile LRS
	Hess et al. (2012)	No significant differences in LRS of hatchery‐origin compared to natural‐origin fish, but trends for lower LRS for hatchery‐origin males
	Anderson et al. (2013)	Hatchery‐origin males demonstrated nonsignificant trends for lower LRS than natural‐origin males, but LRS for females varied between years. Larger males and females demonstrated trends toward higher LRS. Fish that arrived early, compared to later, to the spawning grounds had higher LRS, but in some years, LRS was highest at intermediate dates
	Evans et al. (2015)	Sex, release date, and the interaction between the two significantly predicted LRS. The effect of origin was close to significant with hatchery‐origin fish exhibiting lower fitness than natural‐origin fish
	Evans et al. (2019)	Males and females released earlier had higher LRS. Selection on release date depended on year. Found low but detectable heritability and evolvability estimates for LRS
	Janowitz‐Koch et al. (2019)	Origin, return year, and body length were significant predictors of LRS for both males and females. Return day was significant for males, but not females, indicating that earlier‐returning males demonstrated higher LRS; however, there were trends for disruptive selection on migration timing in both sexes. Trend toward lower LRS for hatchery‐origin males, but most years were not significant for males or females. No significant differences in LRS for crosses containing a hatchery‐origin parent
Coho Salmon (*Oncorhynchus* *kisutch*)	Ford et al. (2006)	No significant differences in LRS between hatchery‐ and natural‐origin fish. Evidence for stabilizing selection on migration timing. Included additional estimates of adult‐to‐juvenile LRS
	Thériault et al. (2011)	Lower LRS of hatchery‐origin compared to natural‐origin males and females. Two‐year‐old jacks (i.e., sneaker males) did not show lower hatchery‐origin compared to natural‐origin LRS. Results were dependent on year
	Kodama et al. (2012)	Evidence for selection on migration timing and size that differed by year, sex, and age. Precipitation related to annual differences
	O’Malley et al. (2015)	No effect of LRS on origin, migration time, or length for any of the cross types. An association between greater number of alleles shared at three immune‐relevant gene markers and increased LRS was found for crosses containing a natural‐origin fish, but not crosses containing two hatchery‐origin fish
Pink Salmon (*Oncorhynchus* *gorbuscha*)	Dickerson et al. (2005)	Higher LRS for males that had higher dominance scores, longer lived, and migrated earlier. Some evidence for stabilizing selection on arrival time and length for females
Sockeye Salmon (*Oncorhynchus* *nerka*)	Peterson et al. (2014)	Dispersers from beach habitat to stream habitat (i.e., immigrants) had significantly lower LRS than fish spawning in their natal stream or spawning in another stream
Steelhead Trout (*Oncorhynchus* *mykiss*)	Kostow et al. (2003)	Lower LRS of hatchery‐origin compared to natural‐ origin fish. Included additional estimates of adult‐to‐juvenile LRS
	McLean et al. (2003), McLean et al. (2004), McLean et al. (2007)	Hatchery‐origin females demonstrated lower LRS than natural‐origin females. Hatchery females that were spawned earlier in the season had significantly higher LRS, but there was no effect on spawning date for hatchery males. No relationship between LRS and female or male length.
	Araki, Ardren, et al. (2007), Araki, Cooper, et al. (2007, 2009)	Evaluated LRS in both a traditional hatchery system with no natural‐origin fish incorporated into a nonlocal broodstock and an integrated supplementation program with a designated proportion of natural‐origin fish incorporated into broodstock. Hatchery‐origin fish (both from a summer and a winter stock) demonstrated lower LRS than natural‐origin fish if the fish originated from the traditional hatchery program. No differences were observed in hatchery‐origin fish from the supplementation program. Compared the LRS between natural‐origin females that crossed with either natural‐origin males, traditional hatchery‐origin males, or supplementation hatchery‐origin males and found no statistically significant effect of male type; trend for crosses involving traditional hatchery‐origin males to have lower LRS than crosses involving natural‐origin males. Compared the LRS between crosses involving fish with different levels of captive breeding in the previous generation. Found a decline in LRS for each successive generation reared in captivity; fish with two hatchery‐origin parents had significantly lower LRS than a fish with two natural‐origin parents; fish with only one hatchery‐origin parent had intermediate, but nonsignificant, LRS between crosses involving two hatchery‐origin parents and those involving zero hatchery‐origin parents.
	Seamons et al. (2007)	Evidence for directional selection for larger body length across sexes and years (although some variation still existed). Evidence for directional selection on early male arrival date, but overall arrival date varied across years for both sexes, providing evidence of stabilizing selection.
	Berntson et al. (2011)	Lower LRS of hatchery‐origin females and males. Generalized linear models (GLMs) showed that LRS was significantly affected by return date, length, origin, and the number of same‐sex competitors. Larger and earlier‐returning fish demonstrated higher LRS, but there was also evidence for stabilizing selection on return date. Included additional estimates of adult‐to‐juvenile LRS.
	Christie et al. (2012)	For the majority of run years, individuals from larger broodstock families demonstrated lower LRS than those producing fewer offspring (<5 offspring). Hatchery‐origin broodstock (1 generation in captivity) outperformed wild‐origin broodstock (no generations in captivity). Adaptation to captivity can occur over one generation with a trade‐off between high performance in captivity and subsequently low performance in the wild.
	Christie et al. (2018)	Estimated LRS from both repeat‐spawning and single‐spawning hatchery‐ and natural‐origin Steelhead Trout. The majority of repeat‐spawning females increased in both length and weight between their first and second spawning. Repeat spawners were more likely natural‐origin compared to hatchery‐origin. Natural‐origin repeat spawners of both sexes had more than 2.5 times the number of offspring than single spawners. First‐time repeat spawners had lower LRS than same‐age single spawners, but higher LRS than same‐age single spawners during the second spawning event, suggesting a trade‐off between reproduction and survival. For single‐spawning fish, LRS increased with male age and size, but for females, there was evidence for negative frequency‐dependent selection on age.

Note

Terms used to describe general findings follow those used in each paper. Within each species, studies are presented first by order of publication date and second by alphabetical order.

Abbreviation: LRS, lifetime reproductive success.

**TABLE 2:** Summary of studies estimating lifetime reproductive success (adult‐to‐juvenile) across species

Species	Reference	General findings
Atlantic Salmon (*Salmo salar*)	Garant et al. (2001)	No relationship between size and LRS for males or females, but there was a significant relationship between number of mates and LRS
	Landry et al. (2001)	There was no evidence for inbreeding avoidance. Mating patterns appeared random overall. Mates chose each other to increase heterozygosity and increase immune defenses of their offspring, as determined by significant differences in the amino‐acid composition of the MHC peptide‐binding region between partners
	Evans et al. (2011)	Parental‐pairs with intermediate MHC amino acid divergence values demonstrated the highest LRS
	Milot et al. (2013)	Hatchery‐origin males and females had lower LRS than natural‐origin fish
	Richard et al. (2013)	LRS positively correlated with number of mates. Effect of catch & release on the number of offspring was size dependent (the LRS of larger fish was more affected than smaller ones). Found an interaction between water temperature and air exposure time on LRS
	Tentelier et al. (2016)	LRS was affected by body size, male tactic, and the interaction between the two. Larger anadromous males had higher LRS than smaller males, but the opposite effect was observed for precocious males
	Mobley et al. (2019)	Both females and males that return to their natal spawning grounds had higher LRS compared to strays (termed “dispersers”). Higher LRS for males and females with older sea age at maturity
	Prévost et al. (2020)	Number of mates significantly predicted LRS among adults that successfully reproduced in one of the two tributaries examined. Both males and females that mated with two or more partners had higher LRS than individuals that only mated with one partner. Body mass, sex, and arrival date were not significant predictors of LRS
	Mobley et al. (2020)	Negative relationship between LRS and freshwater age for females, but not males. Positive relationship between LRS and sea age for both males and females. More time spent in juvenile freshwater habitat was associated with reduced LRS for females but not males. More time spent in marine habitat was associated with increased LRS for males and females. Overall, younger freshwater age was significantly related to older sea age and subsequent increased LRS for females but not males
Brook Charr (*Salvelinus fontinalis*)	Thériault et al. (2007)	Higher LRS for larger females but no effect of size for males. Higher LRS for anadromous compared to resident fish, particularly for females
Brook Trout (*Salvelinus fontinalis*), White‐spotted Charr (*Salvelinus leucomaenis*), and their hybrids	Fukui et al. (2018)	Found a significant positive effect of body size on LRS in males and females. Hybrids of both sexes had lower LRS compared to the parent species. Caudal peduncle depth did not affect LRS
Brown Trout (*Salmo trutta*)	Dannewitz et al. (2004)	The number of mates was significantly associated with male, but not female LRS. Trends for lower LRS in hatchery‐ compared to natural‐origin males. Utilized an artificial stream environment
	Serbezov et al. (2010)	Higher LRS for larger males and females. Evidence for sexual selection on body size
Chinook Salmon (*Oncorhynchus tshawytscha*)	Garner et al. (2009)	Male–female aggression, but not male–male aggression, was correlated with higher male LRS. Female body size, female–female aggression, or female–male aggression did not predict female LRS
	Berejikian et al. (2010)	Nonjack male LRS increased with increasing jack frequency on the spawning grounds. Utilized replicate spawning channels
	Schroder et al. (2010)	Male LRS increased with body weight, attack counts, and courting frequency and decreased with the time spent on the spawning grounds. No differences in hatchery‐ and natural‐origin LRS. Utilized an artificial stream
	Williamson et al. (2010)	Size and age positively affected male LRS while age affected female LRS. Earlier returns had higher LRS. Spawning location impacted LRS. Hatchery‐origin fish were younger on average and had significantly lower LRS
	Evans et al. (2012)	Both male courting and dominance were positively associated with LRS. Male body size was negatively associated with LRS, but there was no relationship between body size and LRS for females. LRS was not affected by intersexual aggression or dominance in females. Aggression toward females did not significantly predict LRS
	Ford et al. (2015)	Naturally spawning resident, early‐maturing, hatchery‐origin males from a captive broodstock program likely represented a large proportion of missing male parents. Anadromous male LRS was significantly higher than LRS of resident males. In general, females spawned with a single anadromous male and multiple resident males, which demonstrated lower per‐capita spawning success than the anadromous males
	Sard et al. (2015)	Hatchery‐origin males (not females) had lower LRS than natural‐origin males, but origin was not significant after accounting for body length. Overall positive relationship between length and LRS for both sexes. No effects of release date or location
Coho Salmon (*Oncorhynchus* *kisutch*)	Neff et al. (2015)	Hatchery‐origin males had lower LRS than natural‐origin males, as did lower‐ranking males. Utilized replicate experimental streams
European Grayling (*Thymallus thymallus*)	Haddeland et al. (2015)	Males and females with a greater number of partners demonstrated higher LRS. Positive relationship for body size and LRS for males, but a negative relationship between body size and LRS for females
Steelhead Trout (*Oncorhynchus* *mykiss*)	Seamons et al. (2004a)	LRS results were dependent on year. Weak relationship between LRS and both maternal and paternal size and to paternal arrival date, but the results were not significant
	Berejikian et al. (2005)	No effect of male or female body mass on LRS. Water current velocity had no effect on LRS. Utilized artificial spawning channels
	Ford et al. (2016)	Migration time, age, size, and spawning location significantly predicted LRS. Positive relationship between size and LRS for both sexes. Evidence for higher LRS for early arriving males, but the relationship between arrival and LRS was complex for both sexes and varied across years. Fish with more hatchery ancestry generally had lower LRS
	Berejikian et al. (2020)	Rearing of males but not females for 1 year in the hatchery significantly increased LRS compared to rearing for 2 years. Utilized artificial spawning channels
Summer Chum Salmon (*Oncorhynchus* *keta*)	Berejikian et al. (2009)	No significant differences in LRS between hatchery‐ and natural‐origin fish. Higher LRS for bigger males. Utilized an artificial stream environment
Westslope Cutthroat Trout (*Oncorhynchus* *clarkii lewisi*)	Muhlfeld et al. (2009)	Length did not affect female LRS but had a small statistically significant effect on male LRS. Increasing admixture with non‐native rainbow trout decreased LRS for males and females
Yellowstone Cutthroat Trout (*Oncorhynchus* *clarkii bouvieri*)	Roth et al. (2019)	Air exposure (simulated to act as catch‐and‐release angling) treatment had no statistically significant effect on male or female LRS

Note

Terms used to describe general findings follow those used in each paper. Within each species, studies are presented first by order of publication date and second by alphabetical order.

Abbreviation: LRS, lifetime reproductive success.

BOX 2 **Common and scientific names of the primary salmonid species from the adult‐to‐adult lifetime reproductive success studies presented in this review, including a description of life‐history forms. Species are presented in alphabetical order**

Atlantic Salmon (*Salmo salar*)—anadromous; iteroparous.

Chinook Salmon (*Oncorhynchus tshawytscha*)—anadromous; semelparous.

Coho Salmon (*Oncorhynchus kisutch*)—anadromous; semelparous.

Pink Salmon (*Oncorhynchus gorbuscha*)—anadromous; semelparous.

Sockeye Salmon (anadromous *Oncorhynchus nerka*)—anadromous form of Kokanee Salmon; semelparous.

Steelhead Trout (anadromous *Oncorhynchus mykiss*)—anadromous form of Rainbow Trout (resident); capable of iteroparity.

## 2 THE IMPACT OF HATCHERY ORIGIN AND CAPTIVE REARING ON LIFETIME REPRODUCTIVE SUCCESS

Understanding the effects of captive breeding is critical to the maintenance and perseverance of species that are of conservation concern (Williams & Hoffman, 2009). An essential component of numerous captive breeding programs is to minimize genetic adaptation to captivity and to confidently estimate the success of reintroducing captive‐born animals back into their species range (Fischer & Lindenmayer, 2000; Frankham, 2008). Due primarily to the conservation status of multiple species of salmonids, the differences between fish born to parents that spawned in nature (i.e., natural‐origin) and those fish born in a hatchery setting from broodstock parents (i.e., hatchery‐origin) have been extensively studied and reviewed in other papers (Araki et al., 2008; Christie et al., 2014; Naish et al., 2007). As such, we provide a brief summary of findings on the effects of origin on LRS and we defer to previously published review papers for further details on this topic, but also account for additional papers that have been published more recently than those reviews.

**FIGURE 1:**
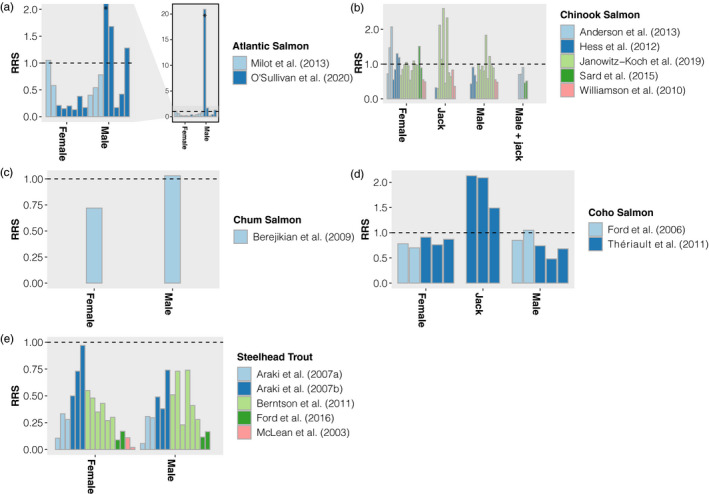
Plot of the relative lifetime reproductive success (LRS) between hatchery‐ and natural‐origin fish for (a) Atlantic Salmon, (b) Chinook Salmon, (c) Chum Salmon, (d) Coho Salmon, and (e) Steelhead Trout across both adult‐to‐juvenile and adult‐to‐adult studies. Studies include those that separately analyzed relative reproductive success (RRS) for males, females, jacks, or jacks and males combined. Each bar represents one estimated value of RRS of each species and sex for each study. Multiple bars can be represented for each study as they reflect a different estimate for each year and are presented in order of year. Dotted line represents equal LRS between hatchery‐ and natural‐origin fish. Due to an outlier in O'Sullivan et al. (2020) (see asterisk), the Atlantic Salmon figure (a) includes an inset representing the correct scale on the y‐axis. Araki, Ardren, et al. (2007) results from the traditional hatchery programs with F1 fish originating from two hatchery‐origin parents and not adjusted for angling harvest. Araki, Cooper, et al. (2007) results from the supplementation program with F1 fish originating from two natural‐origin parents and not adjusted for angling. Berntson et al. (2011) and Ford et al. (2006) results represent adult‐to‐adult RRS. Ford et al. (2016) results from hatchery‐origin fish originating from two hatchery‐origin parents. Hess et al. (2011) and Janowitz‐Koch et al. (2019) results represent RRS estimates that include potential parents producing zero adult offspring. McLean et al. (2003) RRS estimates generated from LRS estimates of Table 3 from Mclean et al. (2003). Williamson et al. (2010) RRS results from offspring collected at the yearling stage from 4‐ and 5‐year‐old (adult) females combined, 4‐year‐old (adult) males, and 3‐year‐old (jack) males separately

Based on author interpretations across salmonid studies and our examination of estimates of hatchery‐origin compared to natural‐origin fish LRS (i.e., relative reproductive success; RRS; relative LRS, or relative fitness), we found trends that hatchery‐origin fish consistently demonstrated lower LRS than natural‐origin fish across species (Figure 1; Anderson et al., 2013; Berntson et al., 2011; Evans et al., 2015; Ford et al., 2016; Kostow et al., 2003; McGinnity et al., 2003; McLean et al., 2003, 2004; Milot et al., 2013; Neff et al., 2015; O'Sullivan et al., 2020; Sard et al., 2015; Thériault et al., 2011; Williamson et al., 2010) as discussed in previous reviews (Araki et al., 2008; Christie et al., 2014; Naish et al., 2007). However, previous research suggests that relative LRS between hatchery‐ and natural‐origin fish is dependent on the type of hatchery or supplementation program under examination. Results in Steelhead Trout (Araki et al., 2007, 2009; Araki et al., 2007; Christie et al., 2012) and Atlantic Salmon (McGinnity et al., 2003, 2004; Mobley et al., 2019) suggest that differences between hatchery‐ and natural‐origin fish in certain systems could, in part, be attributed to hatchery‐origin fish originating from nonlocal origin broodstock and maintained as part of a segregated hatchery system. While reduction of fitness has been documented after a single generation of captive rearing (Araki et al., 2008; Milot et al., 2013), genetic divergence of hatchery strains is expected to be much more rapid in segregated programs with multiple generations of spawning hatchery‐origin fish compared to integrated programs that incorporate natural‐origin fish (Ford et al., 2016; Paquet et al., 2011; Waters et al., 2015). Further, adaptation to captivity can occur in very few generations (Christie et al., 2012), so repeated generations of hatchery rearing would be expected to strengthen domestication selection without input from natural‐origin stocks as predicted by previous models (Baskett & Waples, 2013; Ford, 2002). Similarly, the length of time that hatchery‐origin fish are reared in a hatchery setting may negatively affect LRS (Berejikian et al., 2020). Studies in integrated programs have found little to no significant differences in LRS of crosses containing a hatchery‐origin parent compared to those containing two natural‐origin parents (Ford et al., 2012; Hess et al., 2012), even after two generations (Janowitz‐Koch et al., 2019), providing evidence that there was no reduction in LRS for natural‐origin fish that spawn with hatchery‐origin fish. However, it also worth noting that in integrated programs that aim to incorporate hatchery‐origin fish into the naturally spawning population, RRS estimates may become upwardly biased due to overall reductions in long‐term fitness of the whole population as the proportion of hatchery ancestry increases over time (Willoughby & Christie, 2017). Thus, hatchery programs must carefully weigh goals for conservation versus production when considering spawning and rearing protocols that can lead to varying degrees of hatchery ancestry and domestication.

A second pattern that was detected in our review indicated that differences in LRS for hatchery‐origin fish are influenced by precocial males (e.g., jacks) that tend to have low LRS. Across salmonid studies that analyzed precocial males separately, the difference between hatchery‐ versus natural‐origin male and hatchery‐ versus natural‐origin female LRS appeared smaller (e.g., Hess et al., 2012; Janowitz‐Koch et al., 2019; Thériault et al., 2011; Williamson et al., 2010), and in some cases, female relative LRS was lower than male relative LRS overall (Figure 1; Hess et al., 2012; Janowitz‐Koch et al., 2019). These trends could be the result of different selective pressures on males that adopt a sneaker strategy compared to males that compete for access to females (Thériault et al., 2011). These trends also suggest that inclusion of precocial males across salmonid species can affect estimates of relative LRS, and when possible, the addition of a separate analysis for precocial males could help to determine the effect on LRS patterns in a population. Overall, given the wide range of life‐history variation across salmonids (e.g., resident/anadromous migration, semelparity/iteroparity, premature/mature migration, age at maturity, age at juvenile emigration), disentangling domestication selection due to captive rearing rather than unintentional artificial selection due to inability to account for complex life‐history variation and lack of mate choice (Auld et al., 2019) will provide a clearer picture on the effects of captive breeding.

## 3 EFFECTS OF LIFE HISTORY AND PHENOTYPIC VARIATION ON LIFETIME REPRODUCTIVE SUCCESS

### 3.1 Adult migration timing

While animal migration is a well‐documented phenomenon across the taxonomic spectrum, an inherent complexity exists in understanding how best to manage species of conservation concern with a geographic range that encompasses multiple habitats (Milner‐Gulland et al., 2011). Anadromous salmonids can migrate extensive distances, upward of thousands of kilometers, to oceanic feeding areas and back to freshwater breeding sites. Adult migratory phenotypes are generally classified as those fish that migrate early, such as summer‐run Steelhead Trout and spring‐run Chinook Salmon, and those that migrate later, such as winter‐run Steelhead Trout and fall‐run Chinook Salmon (Quinn et al., 2016). The timing of adult migration in salmonids is a complex and critical life‐history component that is influenced by genetic background, developmental thresholds, ecological conditions such as flow regimes and temperature, olfactory cues, and hormonal changes (Dodson et al., 2013; Quinn, 2005; Quinn et al., 2016; Ueda, 2011). Recently, there has been a surge of studies demonstrating a strong association between adult migration timing and the genes *GREB1L* and *ROCK1* across lineages of Chinook Salmon and Steelhead Trout (Hess et al., 2016; Micheletti & Narum, 2018; Narum et al., 2018; Prince et al., 2017; Thompson et al., 2019, 2020) with genotypic differences that could have differential effects on LRS (Koch & Narum, 2020). Previous research has also suggested that adult migration timing is heritable and can quickly respond to selection (Carlson & Seamons, 2008; Evans et al., 2019; Morita, 2019; Quinn et al., 2000, 2002). Thus, understanding the relationship between adult migratory timing and fitness may have profound conservation implications particularly for salmonids that have experienced differential selection on migration timing (Thompson et al., 2019).

The term “migration timing” is frequently used interchangeably with the terms “run timing,” “arrival timing,” and “return timing”; however, these terms may describe distinct phenotypes that refer to different time points in the migratory trajectory, including entry into freshwater, a common passage point along the migratory corridor, and/or arrival onto the spawning grounds (Quinn et al., 2016). Therefore, we use the general term “migration timing” throughout this manuscript to refer to sampling date at a common migration location but provide clarification on specific migratory phenotypes where appropriate.

General trends in the literature suggest fish that migrate earlier to the spawning grounds demonstrate higher LRS compared to those that arrive later (Table 3; Anderson et al., 2013; Berntson et al., 2011; Dickerson et al., 2005; Ford et al., 2016; Janowitz‐Koch et al., 2019; Kodama et al., 2012; Seamons et al., 2007; Williamson et al., 2010). Previous work in salmonids has demonstrated the adaptive significance and trade‐offs for early, compared to late, migration time for both males and females (Hendry et al., 1999; Morbey, 2000, 2002; Morbey & Ydenberg, 2003; Quinn et al., 2009, 2016). Our review of the literature continues to support this finding across species, where males and females that returned earlier to the spawning grounds generally had positive LRS effects compared to those that arrived later.

**TABLE 3:** Summary of studies that examined the overall relationship between adult migration timing and lifetime reproductive success

Species	Citation	Sex	Data type	Years examined	Type of metric	Value
Chinook Salmon (*Oncorhynchus tshawytscha*)	Williamson et al. (2010)	Female	Adult‐to‐juvenile	2004	GLM estimated model coefficients	−0.15*
	Williamson et al. (2010)	Female	Adult‐to‐juvenile	2005	GLM estimated model coefficients	−0.15*
	Williamson et al. (2010)	Male	Adult‐to‐juvenile	2004	GLM estimated model coefficients	−0.09*
	Anderson et al. (2013)	Male	Adult‐to‐adult	2003	Linear selection gradients	−0.672*
	Anderson et al. (2013)	Male	Adult‐to‐adult	2003	Linear selection differentials	−0.674*
	Anderson et al. (2013)	Male	Adult‐to‐adult	2003	Quadratic selection gradients	1.27*
	Anderson et al. (2013)	Male	Adult‐to‐adult	2003	Quadratic selection differentials	1.00*
	Janowitz‐Koch et al. (2019)	Male	Adult‐to‐adult	Year accounted for in model	GLM estimated model coefficients	−0.008**
Coho Salmon (*Oncorhynchus* *kisutch*)	Ford et al. (2006)	Sex accounted for in model	Adult‐to‐juvenile (fry)	Year accounted for in model	Linear selection differentials	0.092***
	Ford et al. (2006)	Sex accounted for in model	Adult‐to‐juvenile (smolt)	Year accounted for in model	Linear selection differentials	0.060*
	Ford et al. (2006)	Sex accounted for in model	Adult‐to‐adult	Year accounted for in model	Linear selection differentials	0.076**
	Ford et al. (2006)	Sex accounted for in model	Adult‐to‐juvenile (smolt)	Year accounted for in model	Quadratic selection differentials	−0.081***
	Ford et al. (2006)	Sex accounted for in model	Adult‐to‐adult	Year accounted for in model	Quadratic selection differentials	−0.061**
	Kodama et al. (2012)	Female	Adult‐to‐adult	2007	Quadratic selection gradients	−0.53*
	Kodama et al. (2012)	Male	Adult‐to‐adult	2006	Linear selection gradients	(Age 3) −0.50**
	Kodama et al. (2012)	Male	Adult‐to‐adult	2006	Quadratic selection gradients	(Age 2) 0.77***
Pink Salmon (*Oncorhynchus* *gorbuscha*)	Dickerson et al. (2005)	Male	Adult‐to‐adult	Year accounted for in model	Multiple linear regression estimated model coefficients	0.034*
Steelhead Trout (*Oncorhynchus* *mykiss*)	Seamons et al. (2007)	Male	Adult‐to‐juvenile	Combined across years	Linear selection differentials	−0.088*
	Seamons et al. (2007)	Male	Adult‐to‐juvenile	Combined across years	Linear selection gradients	−0.091**
	Berntson et al. (2011)	Sex accounted for in model	Adult‐to‐adult	Combined across years	GLM estimated model coefficients (linear)	−0.13*
	Berntson et al. (2011)	Sex accounted for in model	Adult‐to‐adult	Combined across years	GLM estimated model coefficients (quadratic)	−0.09*
	Berntson et al. (2011)	Sex accounted for in model	Adult‐to‐juvenile	Combined across years	GLM estimated model coefficients (linear)	0.17***
	Berntson et al. (2011)	Sex accounted for in model	Adult‐to‐juvenile	Combined across years	GLM estimated model coefficients (quadratic)	−0.000821***
	Ford et al. (2016)	Male	Adult‐to‐juvenile	2008 (spring returns)	GLM estimated model coefficients	−0.033***

Note

Only statistically significant results are included (*p < 0.05; **p < 0.01; *** *p* < 0.001). Within each species, studies are presented first by order of publication date and second by alphabetical order. Any additional information pertaining to variation in results reported within a study is provide in parentheses.

While there were strong trends for an advantage of early migration, variation still existed in the overall direction and strength of selection on migration timing. For example, Kodama et al. (2012) used selection gradients to estimate the strength and direction of selection across multiple age classes, sexes, and years. Selection differentials measure the mean phenotype both before and after selection, which can be used as a metric of both strength of direct and indirect selection, while selection gradients measure the strength of direct selection on a trait while removing indirect selection of other traits (Arnold & Wade, 1984; Brodie et al., 1995; Falconer & Mackay, 1996; Lande & Arnold, 1983). Kodama et al. (2012) showed that 2‐year‐old Coho Salmon males (jacks) from one broodyear demonstrated significant evidence for disruptive selection on migration timing (Table 3). However, for 3‐year‐old males, there was evidence for selection favoring early migration timing, and in females, there was evidence for stabilizing selection in one broodyear (Table 3). Similarly, Ford et al. (2006) estimated selection differentials on migration timing in Coho Salmon and found evidence of stabilizing selection on migration timing for both sexes (Table 3). Another study did not find a significant effect of migration timing in jack Coho Salmon males, which may possibly reflect a lack of selection on migration timing in jacks which tend to employ sneak fertilization attempts, rather than direct competition for access to females (O'Malley et al., 2015).

Multiple studies also demonstrated trends for disruptive or stabilizing selection, although the results were nonsignificant in some studies or selection was not directly measured (Anderson et al., 2013; Dickerson et al., 2005; Janowitz‐Koch et al., 2019; Seamons et al., 2007). The observed trends indicate that while early migration is generally favorable, differences in selection pressures on migration timing can vary across years, sexes, and even within different life‐history strategies within the same sex. It is not clear what causes these differences, but some likely mechanisms are yearly changes in spawner density, sex ratio, and environmental factors such as precipitation rates that result in highly dynamic and variable environments that may shift selection pressure.

Finally, another factor that could affect differences in results across studies is time of sampling. As mentioned previously, migration timing is defined in different ways across studies in the salmonid literature, with some measuring date of entry into freshwater and others measuring arrival onto the spawning grounds. These differences could be strongly affected by shifts in energy investment and time‐dependent mortality (Hearsey & Kinziger, 2015; Quinn et al., 2016), which could have equally strong effects on estimates of LRS. While most of the studies addressed in this section recorded migration timing as the date of passage at a weir downstream of the primary spawning grounds, variation in actual spawning date likely varies and remains unaccounted for in these studies which could be shaping differences in LRS values across studies.

### 3.2 Effects of age and size

Body size is a ubiquitous trait across the animal kingdom that correlates with numerous physiological, ecological, and life‐history processes and is driven by both sexual and natural selection (Andersson, 1994; Berns, 2013; Blackburn & Gaston, 1994). Variation in body size in salmonids can have important ecological implications throughout various life‐history stages, particularly for females which have a demonstrated strong positive relationship between body size and fecundity, competitive advantages on the spawning grounds, and an overall increase in nest quality (Berghe & Gross, 1984; Fleming & Gross, 1994; Fleming & Reynolds, 2004; Hixon et al., 2014). For males, large body size is generally correlated with an increase in sperm output and a subsequent increase in the likelihood of successful fertilization, increased intrasexual competitive advantages on the spawning grounds, and increased intersexual behaviors, such as courtship, on the spawning grounds (Bolgan et al., 2017; Thomaz et al., 1997; Watanabe et al., 2008). In addition, results from behavioral studies suggest that both male and female salmonids demonstrate consistent trends for preferring mates with larger body size, even in the absence of direct benefits (Auld et al., 2019).

Mating preferences and reproductive benefits for large size (Auld et al., 2019; Fleming & Reynolds, 2004) were clearly reflected in our review of LRS in salmonids, where body size generally demonstrated a positive relationship with LRS (Table 4; Anderson et al., 2013; Berejikian et al., 2009; Berntson et al., 2011; Ford et al., 2016; Fukui et al., 2018; Haddeland et al., 2015; Janowitz‐Koch et al., 2019; Muhlfeld et al., 2009; Sard et al., 2015; Schroder et al., 2010; Seamons et al., 2007; Serbezov et al., 2010; Tentelier et al., 2016; Thériault et al., 2007; Williamson et al., 2010). However, the strength and direction of selection on size was sometimes dependent on sex and year (Table 4; Kodama et al., 2012). Age, which is generally correlated with size, also demonstrated a positive relationship with LRS in some studies (Mobley et al., 2019, 2020), but the results were sex‐specific in another study which demonstrated a positive relationship in males, but a more complex relationship in females (Christie et al., 2018).

**TABLE 4:** Summary of studies that examined the overall relationship between body size and lifetime reproductive success

Species	Citation	Sex	Data type	Years examined	Type of metric	Value
Atlantic Salmon (*Salmo salar*)	Tentelier et al. (2016)	Female	Adult‐to‐juvenile	Combined across years	Linear regression (coefficient t‐value)	3.98**
	Tentelier et al. (2016)	Male	Adult‐to‐juvenile	Combined across years	GLM estimated model coefficients (negative binomial; anadromous males)	0.55*
Brook Charr (*Salvelinus fontinalis*)	Thériault et al. (2007)	Female	Adult‐to‐juvenile	Combined across years	Coefficient of determination	0.46***
Brook Trout (*Salvelinus fontinalis*), White‐spotted Charr (*Salvelinus leucomaenis*), and their hybrids	Fukui et al. (2018)	Female	Adult‐to‐juvenile	Combined across years	GLM estimated model coefficients	5.4987***
	Fukui et al. (2018)	Male	Adult‐to‐juvenile	Combined across years	GLM estimated model coefficients	0.0147***
Brown Trout (*Salmo trutta*)	Serbezov et al. (2010)	Sex accounted for in full model	Adult‐to‐juvenile	Combined across years	GLM estimated model coefficients	0.02***
Chinook Salmon (*Oncorhynchus tshawytscha*)	Schroder et al. (2010)	Male	Adult‐to‐juvenile	Combined across years	Coefficient of determination	0.26***
	Williamson et al. (2010)	Female	Adult‐to‐juvenile	2004	GLM estimated model coefficients	0.20*
	Williamson et al. (2010)	Female	Adult‐to‐juvenile	2005	GLM estimated model coefficients	0.09*
	Williamson et al. (2010)	Male	Adult‐to‐juvenile	2004	GLM estimated model coefficients	0.79*
	Williamson et al. (2010)	Male	Adult‐to‐juvenile	2005	GLM estimated model coefficients	0.27*
	Anderson et al. (2013)	Female	Adult‐to‐adult	2003	Linear selection gradients	0.749*
	Anderson et al. (2013)	Female	Adult‐to‐adult	2004	Linear selection gradients	0.422**
	Anderson et al. (2013)	Female	Adult‐to‐adult	2005	Linear selection gradients	0.810*
	Anderson et al. (2013)	Female	Adult‐to‐adult	2003	Linear selection differentials	0.638*
	Anderson et al. (2013)	Female	Adult‐to‐adult	2004	Linear selection differentials	0.422**
	Anderson et al. (2013)	Female	Adult‐to‐adult	2005	Linear selection differentials	0.670*
	Anderson et al. (2013)	)Female	Adult‐to‐adult	2005	Quadratic selection gradients	2.55**
	Anderson et al. (2013)	Female	Adult‐to‐adult	2005	Quadratic selection differentials	1.23***
	Anderson et al. (2013)	Male	Adult‐to‐adult	2003	Quadratic selection gradients	−1.02*
	Sard et al. (2015)	Sex accounted for in full model	Adult‐to‐juvenile	2011	GLM estimated model coefficients	0.055***
	Janowitz‐Koch et al. (2019)	Female	Adult‐to‐adult	Year accounted for in model	GLM estimated model coefficients	0.030**
	Janowitz‐Koch et al. (2019)	Male	Adult‐to‐adult	Year accounted for in model	GLM estimated model coefficients	0.034**
Coho Salmon (*Oncorhynchus* *kisutch*)	Kodama et al. (2012)	Female	Adult‐to‐adult	2006	Linear selection gradients	0.30*
	Kodama et al. (2012)	Female	Adult‐to‐adult	2007	Quadratic selection gradients	−0.63**
	Kodama et al. (2012)	Male	Adult‐to‐adult	2007	Quadratic selection gradients	(Age 3) 0.83***
European Grayling (*Thymallus thymallus*)	Haddeland et al. (2015)	Female	Adult‐to‐juvenile	2008	GLM estimated model coefficients	*χ*^2^ = 5.6*
	Haddeland et al. (2015)	Male	Adult‐to‐juvenile	2008	GLM estimated model coefficients	*χ*^2^ = 36.8***
Steelhead Trout (*Oncorhynchus* *mykiss*)	Seamons et al. (2007)	Female	Adult‐to‐juvenile	Combined across years	Linear selection differentials	0.074*
	Seamons et al. (2007)	Female	Adult‐to‐juvenile	Combined across years	Linear selection gradients	0.076*
	Seamons et al. (2007)	Male	Adult‐to‐juvenile	Combined across years	Linear selection differentials	0.096**
	Seamons et al. (2007)	Male	Adult‐to‐juvenile	Combined across years	Linear selection gradients	0.09**
	Berntson et al. (2011)	Sex accounted for in model	Adult‐to‐adult	Date accounted for in model	GLM estimated model coefficients	0.09*
	Berntson et al. (2011)	Sex accounted for in model	Adult‐to‐juvenile	Date accounted for in model	GLM estimated model coefficients	0.0018*
	Ford et al. (2016)	Female	Adult‐to‐juvenile	2009	GLM estimated model coefficients	0.058***
	Ford et al. (2016)	Female	Adult‐to‐juvenile	2010	GLM estimated model coefficients	0.033*
	Ford et al. (2016)	Female	Adult‐to‐juvenile	2011	GLM estimated model coefficients	0.044**
	Ford et al. (2016)	Male	Adult‐to‐juvenile	2008	GLM estimated model coefficients	0.133***
	Ford et al. (2016)	Male	Adult‐to‐juvenile	2009	GLM estimated model coefficients	0.122***
	Ford et al. (2016)	Male	Adult‐to‐juvenile	2010	GLM estimated model coefficients	0.094***
	Ford et al. (2016)	Male	Adult‐to‐juvenile	2011	GLM estimated model coefficients	0.075***
Summer Chum Salmon (*Oncorhynchus* *keta*)	Berejikian et al. (2009)	Male	Adult‐to‐juvenile	2004 (East channel)	Coefficient of determination	0.13*
	Berejikian et al. (2009)	Male	Adult‐to‐juvenile	2004 (West channel)	Coefficient of determination	0.19*
	Berejikian et al. (2009)	Male	Adult‐to‐juvenile	2005 (East channel)	Coefficient of determination	0.19*
Westslope Cutthroat Trout (*Oncorhynchus* *clarkii lewisi*)	Muhlfeld et al. (2009)	Male	Adult‐to‐juvenile	Year accounted for in model	GLM estimated model coefficients	0.0065*

Note

Only statistically significant results are included (*p < 0.05; **p < 0.01; *** *p* < 0.001). Within each species, studies are presented first by order of publication date and second by alphabetical order. Any additional information pertaining to variation in results reported within a study is provide in parentheses.

There are a number of potential mechanisms that could shift selection on age and body size across years, particularly for Coho Salmon and Steelhead Trout females (Christie et al., 2018; Kodama et al., 2012). It is possible that the operational sex ratio (the ratio of sexually receptive males to females; Emlen & Oring, 1977), overall breeding density, and competition on the spawning grounds, or predation rates could affect the strength of sexual and natural selection in any given year, resulting in evolutionary stability of multiple sizes (Berejikian et al., 2010; Fleming & Gross, 1994; Holtby & Healey, 1986; Tentelier et al., 2016). Christie et al. (2018) provided evidence that negative frequency‐dependent selection acting on age could be shaping variation in female Steelhead Trout. As such, while it is reasonable to assume that larger body size necessarily translates into a higher reproductive output (i.e., LRS), yearly environmental and/or demographic shifts could potentially affect the strength and direction of selection on body size.

Multiple studies did not find a positive relationship between LRS and body size (Berejikian et al., 2005; Dickerson et al., 2005; Evans et al., 2012; Garant et al., 2001; Garner et al., 2009; McLean et al., 2007; O'Malley et al., 2015; Prévost et al., 2020). However, the results from some of these studies may not be generalizable to natural populations because they tested the effects of body size in only jack males (O'Malley et al., 2015) and used hatchery‐origin fish produced from nonlocal origin and highly domesticated broodstock (McLean et al., 2007), or the experiment was conducted under artificial conditions (Berejikian et al., 2005), for example.

### 3.3 Resident versus anadromous

There have been a limited number of studies comparing LRS differences between resident and anadromous life‐history forms within a population, which are inherently correlated with body size. However, studies noted sex‐specific effects of residency versus anadromy on LRS that provide insight to the maintenance of both life‐history types. One of the most intriguing studies comparing LRS between anadromous versus resident life‐history types in Brook Charr found that anadromous females had higher LRS than resident females, with results driven by the larger size of anadromous compared to resident females (Thériault et al., 2007). The authors posited that the tactic of residency may be beneficial and therefore persist, where small tributary streams are easily accessible to smaller residents but could exclude larger anadromous females (Thériault et al., 2007). In the same study, there were no observed differences in LRS between resident and anadromous males, a potential reflection of opportunistic behavior, such as sneaking by smaller resident males rather than fighting (Thériault et al., 2007). Other studies in Steelhead Trout revealed only a very small number of offspring were assigned to resident parents overall (Berntson et al., 2011), which reflected both logistical constraints regarding sampling resident fish (resulting in a large fraction of missing resident parents) and the overall low assignment success of hatchery‐origin resident fish (Berntson et al., 2011).

### 3.4 Local adaptation

Numerous salmonid species demonstrate high fidelity to natal spawning sites (Hendry et al., 2004; Quinn, 1993). Higher fitness in the local habitat (i.e., natal spawning sites compared to foreign sites in salmonids) can affect spatial distribution and genetic diversity and can promote reproductive isolation and is, therefore, a central theme in animal conservation (Fraser et al., 2011; Kawecki & Ebert, 2004; Savolainen et al., 2013; Taylor, 1991). However, although numerous studies have measured survival differences between salmonids that stray from their natal spawning sites and those that return, very few studies have actually estimated differences in LRS, a key component of adaptive variation and evolution (Fraser et al., 2011; Garcia de Leaniz et al., 2007). Strays among distinct lineages or geographically distant populations are expected to be relatively rare and have low LRS (Hess et al., 2014; Keefer & Caudill, 2014; Quinn, 1993). However, straying among streams within the same drainage is common (Ford et al., 2015; Keefer & Caudill, 2014) and colonization of newly available habitat (Anderson et al., 2013) may be beneficial for preventing extirpation of local populations (Hill et al., 2002).

Further, different ecotypes that occur within the same system may be under selective pressure at temporal or fine geographic scales resulting in outbreeding depression (Gharrett et al., 1999) and introgression (Hess et al., 2011) when ecotypes are interbred artificially, but little is known about LRS among salmonid ecotypes. A study of Sockeye Salmon ecotypes in southwest Alaska found that dispersers from beach habitat to stream habitat (i.e., immigrants) had significantly lower LRS than fish spawning in their natal stream or fish spawning in another stream (Peterson et al., 2014). The authors provided potential mechanisms shaping differences in LRS in this study, including morphological maladaptation and reduced survival of hybrid offspring. Similarly, Atlantic Salmon that were native to spawning grounds demonstrated higher LRS compared to those dispersing from neighboring areas (Mobley et al., 2019). On a broad scale, these results suggest that differences in LRS between immigrant and philopatric fish can serve as a barrier to reduce gene flow between populations and thus further reinforce local adaptation. However, the maintenance of gene flow, even at low levels observed in the Peterson et al. (2014) study, can still promote adaptive genetic diversity within populations, a key component of salmonid evolution, persistence, and conservation policy decisions (Waples, 1991).

## 4 EFFECT OF BEHAVIOR AND SPAWNING INTERACTIONS ON LIFETIME REPRODUCTIVE SUCCESS

### 4.1 Spawning behavior

Animal behavior is a central theme in ecology and evolution that spans across species, involving both intrasexual and intersexual interactions (Alcock, 2001; Dugatkin, 2020). Numerous behavioral factors, such as dominance, courtship, and density, can affect LRS in salmonids. For example, Berntson et al. (2011) used the number of same‐sex competitors on the spawning grounds of Steelhead Trout as a proxy for competition. Berntson et al. (2011) found that the number of same‐sex competitors negatively affected LRS and that females, in particular, were negatively affected by a greater female density on the spawning grounds. Previous research has suggested that breeding density on the spawning grounds is the primary driver of female competition in salmonids with females competing for nest sites, displacing other females, and disturbing nests (i.e., “redds”; Fleming & Gross, 1989; van den Berghe & Gross, 1989). However, while some studies have demonstrated a relationship between spawner density and redd superimposition (Beard Jr & Carline, 1991; Fukushima et al., 1998), others have not (Essington et al., 1998; Gortázar et al., 2012; Peterson et al., 2020).

Aggressive or dominant mating behavior among males on the spawning grounds has also been examined across a limited number of studies, with a general positive relationship of LRS with increasing aggressiveness. Aggressive behaviors in males that have been tied to higher LRS include dominance in courting behaviors (Dickerson et al., 2005; Evans et al., 2012), attack frequency to competitors on spawning grounds (Schroder et al., 2010), and frequency of courting attempts with females (Evans et al., 2012; Schroder et al., 2010). However, effects of aggressive male behavior may diminish later into the spawning season, potentially as a result of a shift in the operational sex ratio that could cause a reduction in the ability of larger males to maintain access to spawning females through a decrease in physical condition over time, the overall completion of female egg deposition, and an increase in the arrival of new spawners (Dickerson et al., 2005; Quinn et al., 1996). A positive relationship between the number of mates and LRS (Dannewitz et al., 2004; Garant et al., 2001; Haddeland et al., 2015; Prévost et al., 2020; Richard et al., 2013) and a negative relationship between the number of days spent on the spawning grounds and LRS (Schroder et al., 2010) have also been documented.

### 4.2 Mate choice

Mate choice is another component of salmonid behavior that can affect LRS (Auld et al., 2019; Fleming & Reynolds, 2004; Petersson & Järvi, 2015). However, parentage‐based studies in salmonids generally have not distinguished between preferred mates and successful mates. Additionally, mating preferences cannot usually be exercised in captive breeding programs of salmonids. Thus, literature on the relationship between LRS and salmonid mate choice is largely underdeveloped. One area of mate choice that has received some attention in salmonid LRS studies is MHC‐dependent mate choice. Across taxonomic groups, MHC‐dependent mate choice through disassortive mating has been shown to optimize genetic compatibility and increase overall pathogen resistance in offspring (Bernatchez & Landry, 2003; Milinski, 2006; Tregenza & Wedell, 2000). In salmonids, general trends in the literature suggest that there are higher levels of population differentiation at MHC genes compared to neutral genes (reviewed in Bernatchez & Landry, 2003). However, there have been very few studies to date that have estimated the effect of MHC‐dependent mate choice on salmonid LRS, with variable results across species. While some studies provided evidence for MHC‐dependent mate choice through disassortative mating in Chinook Salmon (Garner et al., 2009) and Atlantic Salmon (Evans et al., 2011; Landry et al., 2001), another study in Coho Salmon (O'Malley et al., 2015) found that LRS increased with a greater number of shared alleles at two MHC genes and one immune‐related gene. While disassortive mating can provide genetic benefits to offspring, the expression of MHC‐dependent mate choice may be limited by intersexual aggression, presenting a scenario of sexual conflict (Garner et al., 2009). In contrast, O'Malley et al. (2015) hypothesized that the positive effect of increasing shared alleles between females and jack males, which use a sneaker‐male mating strategy, could have evolved as a mechanism to reduce the likelihood of hybridization and outbreeding depression, or as a mechanism to increase the likelihood of specific combinations of MHC alleles that could confer a survival advantage in offspring. Additionally, the strength of the relationship between LRS and MHC genes in this study differed across years, a finding which could be related to changes in the overall density and composition of larger males and jack males on the spawning grounds on a yearly basis (O'Malley et al., 2015). Therefore, the effect of MHC‐dependent mate choice on LRS may be temporally and species‐dependent and affected by the composition of life‐history strategies in the population. Future studies are needed to further explore the relationship between MHC‐dependent mate choice and LRS in salmonids.

### 4.3 Hybridization with non‐native species

Hybridization and introgression with non‐native species of fish with distinct life histories can also affect LRS. In general, hybridization plays an important role in evolution either constraining the evolution of new species or in propelling diversification (Arnold, 1997; Mayr, 1963) and can have important implications for setting conservation policies (Allendorf et al., 2001). For example, in one study, the LRS of native Westslope Cutthroat Trout significantly declined for both males and females with increasing admixture from non‐native Rainbow Trout (Muhlfeld et al., 2009). Likewise, hybrid offspring from introduced Brook Trout and native White‐spotted Charr showed significantly lower LRS compared to their parental species (Fukui et al., 2018).

While there are few studies directly evaluating LRS related to local adaptation in salmonids, results support expectations that native fish that are adapted to local environments have higher fitness than strays or non‐native species. However, this is a rich area in need of further study to better estimate fitness related to local adaptation.

## 5 CAVEATS, FUTURE DIRECTIONS, AND CONSERVATION IMPLICATIONS

### 5.1 Accounting for various life‐history strategies

**FIGURE 2:**
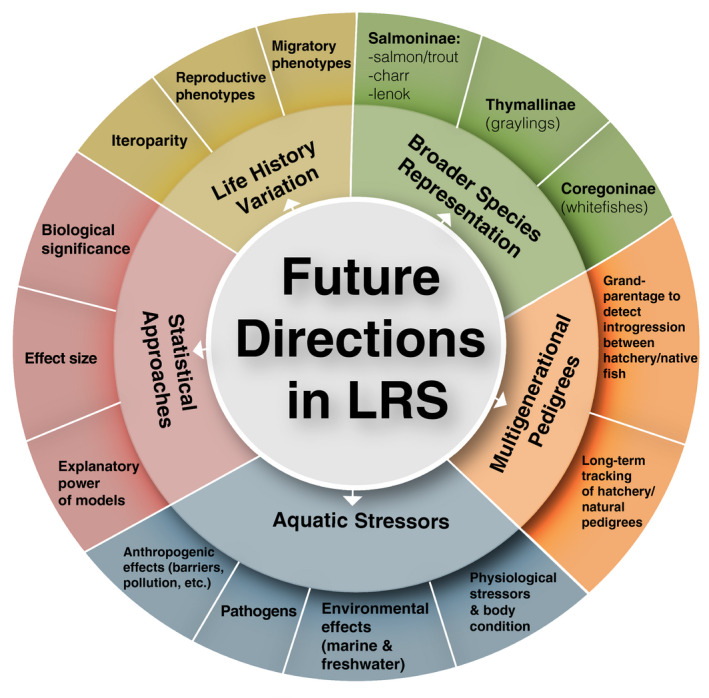
Circular concept map exploring the potential areas of future research in salmonid lifetime reproductive success (LRS) studies. Each color represents a general topic area for future research. Inner ring represents five general topic areas and outer ring represents specific examples of directions for future studies

While the review presented here demonstrated general trends in the factors affecting salmonid fitness, there are numerous areas of research that require further exploration yet are largely absent from the literature (Figure 2). One such example is inclusion of the vast array of salmonid life‐history strategies in LRS studies. Accounting for distinct salmonid life‐history variation may not only help to reduce variability in results across studies but also remains an extremely important component of successful conservation management. For example, we observed here that when comparing hatchery‐origin versus natural‐origin LRS (i.e., RRS), differences in LRS between alternative reproductive phenotypes (precocial males and larger hooknose males) can drive overall results. Therefore, future LRS studies should determine the effect of both including and excluding precocial males in the dataset whenever possible. Other life‐history strategies such as iteroparity, freshwater residency, duration spent in the ocean, and adult migration timing also remain largely unaccounted for in salmonid fitness studies.

Only a limited number of studies in salmonids have examined the differences in LRS between resident and anadromous fish (Berntson et al., 2011; Christie et al., 2011; Ford et al., 2015; Thériault et al., 2007) due to sampling challenges in experimental design. In species with both anadromous and resident forms, sampling efforts have been directed almost exclusively on returning anadromous fish due to low densities and practical challenges of sampling residents, resulting in a large fraction of “missing” resident parents (Araki, Ardren, et al., 2007; Christie et al., 2011; Seamons et al., 2004b). One potential method to circumvent incomplete sampling of resident fish that has recently received attention is to trace anadromous offspring back to their grandparents, essentially filling in the blanks of a pedigree that has complete sampling of anadromous, but not resident, fish (Christie et al., 2011; Ford et al., 2015; Sard et al., 2016).

There have also been very few studies that have examined fitness differences between semelparous and iteroparous life‐history types that occur within the same species (Christie et al., 2018; Seamons & Quinn, 2010). In particular, salmonids that display iteroparity (i.e., repeat spawners) are greatly under‐represented in LRS studies. Since very few studies have been able to account for repeat spawners, it is possible that iteroparous individuals that either minimized energy investment and subsequent reproductive output in the first breeding season and/or failed to reproduce during the second breeding season display disproportionally low LRS compared to the semelparous individuals in the same population, potentially lowering overall population‐level trends of LRS (Seamons & Quinn, 2010). Two studies in Steelhead Trout showed that LRS of iteroparous fish averaged more than twice the LRS of those spawning one time, suggesting that iteroparous fish can increase population abundance overall (Christie et al., 2018; Seamons & Quinn, 2010). Despite logistical challenges of assessing LRS in iteroparous species, increasing the incidence of iteroparous fish within a population has become a tool to increase overall genetic variability and population abundance, particularly for declining populations of Steelhead Trout (Copeland et al., 2019; Hatch et al., 2013; Narum et al., 2008). Therefore, it is important and necessary for the breadth of LRS studies to continue to expand across a wide range of life‐history types, especially as these studies help to inform broad‐scale management and conservation decisions for iteroparous species.

Life‐history traits such as adult migration timing and duration spent in freshwater versus ocean are often variable within salmonid populations (Quinn et al., 2016) but have also largely been unaccounted for in studies of LRS with only a few recent exceptions (Ford et al., 2016; Janowitz‐Koch et al., 2019; Mobley et al., 2020). Given that genes of major effect have been shown to drive phenotypic variation for these traits across species of salmonids (Barson et al., 2015; Prince et al., 2017; Waters et al., 2021), accounting for trait variation can substantially influence estimates of LRS within populations. For example, if hatchery‐origin fish are returning to the spawning grounds later in the season and experiencing reduced LRS due to increasing stream temperatures compared to natural‐origin fish that are returning earlier on average, this could have important conservation management implications that would need to be addressed. There is also increasing interest to investigate LRS of fish that are heterozygous for genes of major effect relative to alternative homozygous fish for phenotypic traits such as adult migration timing where one of the life‐history types is under high conservation concern (i.e., spring‐run Chinook Salmon; Thompson et al., 2019). In general, we expect that future studies of LRS will need thorough study designs that account for life‐history types and traits.

### 5.2 Expanding studies across salmonid species

It is also important to point out that the majority of LRS studies have examined four primary species of Salmoninae—Chinook Salmon, Steelhead Trout, and Atlantic Salmon, followed by Coho Salmon—and substantially fewer studies in other species of Salmoninae, such as Chum Salmon, Sockeye Salmon, Pink Salmon, Charr, and Lenok. Similarly, Thymallinae (Graylings) and Coregoninae (Whitefishes) are also lacking in the literature. Under‐representation of certain species is not necessarily from a lack of interest. Technical constraints, particularly those that are inherent in threatened or endangered species, such as limited sample size, inaccessible habitat, or fishing regulations can limit the ability of sample collection in certain species and populations. Extrapolating results from studies across salmonid species may not sufficiently encompass general reproductive patterns in all salmonid species or even across populations of the same species. In addition, factors that impact LRS, such as origin (hatchery versus natural) may differentially impact certain species compared to others (Araki et al., 2008).

### 5.3 Estimating reproductive success across multiple generations

An important area of research that is beginning to receive more attention in salmonid LRS studies is estimating fitness across multiple generations. However, only a small number of studies have utilized grandparentage in estimating salmonid LRS using either genetic exclusion methods (Christie et al., 2011; Ford et al., 2015; Sard et al., 2016), maximum likelihood approaches (Letcher & King, 2001), or in instances where long‐term pedigree data are available, directly tracing pedigrees over consecutive generations (Janowitz‐Koch et al., 2019). Grandparentage has the potential to provide estimates of long‐term fitness effects, particularly in hatchery‐ versus natural‐origin RRS studies, and further could help to determine levels of introgression between hatchery and native fish. For example, Janowitz‐Koch et al. (2019) provided evidence that LRS did not significantly decline for natural‐origin fish that spawned with hatchery‐origin fish, even after the offspring of these crosses were tracked for two generations. Although very useful and informative, constructing multigenerational pedigrees presents logistical challenges, with some programs not able to sample fish for multiple generations, particularly for longer lived salmonids. In addition to practical challenges, grandparentage assignment requires large and diverse genetic marker panels (Letcher & King, 2001), which have not yet been developed for many salmonid species. With the further development of grandparentage software programs (e.g., Huisman, 2017), the long‐term fitness effects of factors such as captive breeding and anthropogenic impacts for salmonids will be further explored and potentially integrated into management practices.

### 5.4 Exploring the effects of environmental factors and anthropogenic impacts

Numerous studies reviewed here reported large differences in LRS across years (e.g., Anderson et al., 2013; Dickerson et al., 2005; Janowitz‐Koch et al., 2019; Kodama et al., 2012; Sard et al., 2015; Seamons et al., 2004a, 2007; Thériault et al., 2011). There are numerous factors that could explain these interannual differences including ocean conditions (e.g., upwelling, sea surface temperature), freshwater environment at natal sites and through migratory corridors (e.g., water temperature, precipitation rates, changes in water velocity), and dam passage effects (both downstream and upstream) that are likely shaping these differences in fitness and survival (National Research Council, 2004). Water temperature and water flow, in particular, can strongly affect salmonid development, growth, and survival across all stages of the life cycle (Jonsson & Jonsson, 2009; Pankhurst & Munday, 2011). For example, elevated levels of stress and mortality in early‐entry late‐run Sockeye Salmon are directly linked to warm river temperatures that are at, or near, thermal maxima of the species (Hinch et al., 2012). During ocean phases of the life cycle, environmental conditions such as upwelling of cold water can directly affect food chain structure and subsequent growth and survival in salmonids (Bi et al., 2011; Black et al., 2011; Emmett et al., 2006; Fisher & Pearcy, 1988; Peterson & Schwing, 2003; Scheuerell & Williams, 2005). Other ocean‐related factors such as Pacific Decadal Oscillation can affect salmonid ocean abundance and subsequent returns to freshwater (Hare et al., 1999; Mantua & Hare, 2002; Mantua et al., 1997; Peterson et al., 2010).

Environmental variables can also interact with both demographic variables and phenotypic traits, such as density, size, or origin, to predict survival in salmonids (Bowerman et al., 2021; Crozier et al., 2008; Zabel et al., 2006). While we recognize the need for these types of environmental data in studies predicting fitness, we also understand the potential difficulty in obtaining these metrics during field sampling. One potential source for obtaining environmental data is through pre‐existing databases, such as those maintained by federal agencies (e.g., Huang et al., 2017; Isaak et al., 2017) or spatial interpolated climate data across the earth collected from satellites (e.g., Fick & Hijmans, 2017).

Direct anthropogenic impacts are another type of aquatic stressor that can shape patterns in LRS. For example, trace heavy metals, pesticides, and herbicides could all affect survival and fitness in salmonids (Milner et al., 2003; Wedemeyer et al., 1980). The impact of recreational fishing techniques should also be expected to affect LRS, yet studies in the literature are lacking. One example of a recreational fishing technique is “catch and release” (CR), a technique that involves releasing live fish back into the water after catching them. The use of CR, and the subsequent likelihood of mortality, remains controversial from a cultural, ethical, and biological perspective (Arlinghaus et al., 2007). Air exposure during CR has been shown to negatively affect LRS; however, the relationship between LRS and air exposure may be dependent on other factors such as water temperature and size of the fish (Richard et al., 2013). Other studies have shown no effect of air temperature on LRS (Roth et al., 2019); the differences in results potentially attributed to differences in sample sizes. Therefore, the impacts of human activities, such as fishing, on LRS remains an area of research that is largely untested.

### 5.5 The effect of general body condition and pathogens on reproductive success

Numerous infectious diseases and parasites have been extensively documented in salmonids (Bakke & Harris, 1998). However, there have been very few studies measuring the impact of disease on reproductive success, particularly in natural salmonid populations, despite the fact that heritable variation has been documented in these factors and they can directly impact fitness‐related traits, such as growth, feeding behavior, swimming, and osmoregulation (Garcia de Leaniz et al., 2007; Mendel et al., 2018; Miller et al., 2014; Yáñez et al., 2014). While lethal sampling has been employed in numerous studies assessing pathogens in salmonids, other studies have employed nonlethal methods (e.g., Elliott et al., 2015; Fernández‐Alacid et al., 2018; Kittilsen et al., 2009; Rees et al., 2015). The use of noninvasive and nonlethal sampling across salmonid studies remains a priority for state, federal, and tribal fisheries agencies, and much progress has been made on developing these sampling techniques (Coble et al., 2019; Lawrence et al., 2020; Teffer & Miller, 2019). Therefore, an assessment of the effect of pathogens on reproductive success in salmonids is a tractable area of research and remains an area of high need. Similarly, the effect of general physiological condition on reproductive success, particularly an overall assessment of energy reserves, is another area of research that is lacking and recent advances in technology have allowed for noninvasive sampling of body condition in salmonids (Hanson et al., 2010). By including factors such as pathogen load and general body condition, a clearer understanding of the aquatic stressors affecting fitness in salmonids will more fully develop. Of particular importance would be determining whether these factors interact with other factors to predict LRS, such as origin or environmental conditions, which could help within the broader context of successful conservation management.

### 5.6 Explaining variation in results across studies and within studies

While the review presented here demonstrates general patterns and trends of factors affecting LRS, there is a substantial amount of variability in results across studies. One obvious potential source of variability is the vast array of factors, or lack thereof, used to predict LRS across studies. For example, as discussed previously, while many studies estimating differential LRS of hatchery‐ and natural‐origin fish accounted for body size and/or age (Anderson et al., 2013; Berntson et al., 2011; Ford et al., 2012, 2016; Janowitz‐Koch et al., 2019; Sard et al., 2015; Thériault et al., 2011; Williamson et al., 2010), several others did not, which could greatly impact the overall results of these types of studies. Several studies provided evidence that hatchery‐origin fish in some years were smaller than natural‐origin, which may, in part, help to explain the differences in LRS that have been observed between hatchery‐ and natural‐origin fish. For male salmonids, while some studies have found that hatchery‐origin precocial males may not experience the same fitness declines as hatchery‐origin males of older age classes (Garant et al., 2003; Thériault et al., 2011), other studies show opposite effects, with hatchery‐origin precocial males exhibiting significantly lower LRS than natural‐origin precocial males (Hess et al., 2012; Janowitz‐Koch et al., 2019). These results provide a clear example that age and/or size can interact with origin to predict fitness, and both should be accounted for in these types of studies whenever possible. However, it is also worth noting that including additional factors in models may not necessarily explain additional variability in salmonid LRS studies.

One method to determine assessment of model fit in a study is estimating the proportion of variation in the response variable that can be explained by the model. However, out of the numerous studies that we reviewed here, only a small number of studies provided estimates on proportion of variability in LRS explained by models. For example, the total variance in LRS models (as reported by the coefficient of determination estimates, *R* ^2^) explained by body size was the only trait that was consistently reported for a few studies, yet exhibited marked variation ranging from *R* ^2^ < 2% (Seamons et al., 2007) to 46% (Thériault et al., 2007) for female Steelhead Trout and female Brook Charr, respectively. For males, variation explained by body size was also variable, ranging from <3% in Steelhead Trout (Seamons et al., 2007) to 26% in Chinook Salmon (Schroder et al., 2010) with results for Chum Salmon males somewhere in between (approximately 13%–19%; Berejikian et al., 2009). Similarly, the proportion of variation on LRS explained by migration timing was only provided in a single study (Seamons et al., 2007). Although major conclusions cannot be drawn across the limited number of studies provided here, the percent of variation in traits explaining LRS models can provide important statistical and biological information within studies that could perhaps be generalizable to similar study systems.

It is also possible that general differences in model results both within studies (across years) and between studies could be the result of populations experiencing different types and varying strength of selection, sometimes experiencing weak selection and residing in a stable optimum, other times facing strong directional selection and shifting optima, for example. Selection fluctuations can be caused by numerous factors in salmonids, including environmental variables, changing frequencies of life‐history forms, sex ratio, competition, and overall spawner density, to name a few (Anderson et al., 2010; Christie et al., 2018; Dickerson et al., 2005; Ford et al., 2008; Kodama et al., 2012; Seamons et al., 2007). These factors are likely to shift within populations across years, which could explain the variability in results seen across years within the same study. Similarly, these factors would be expected to be different across populations with varying geographical landscapes and different genetic and historical backgrounds. Therefore, differences across studies in traits predictive of fitness or a lack of significance in certain traits that should be expected to predict fitness, such as body size, could also be the result of differences in temporal or spatial selective pressures.

### 5.7 Biological significance and effect sizes

Although we provide compelling evidence that numerous demographic, phenotypic, phenological, environmental, and behavioral factors predict fitness, we rarely uncovered a discussion of biological significance in literature reviews. While statistical significance is an important part of drawing experimental conclusions, it is only one component of biological significance. Biological significance is broadly defined as a biological effect, or the size of a biological effect, that is biologically meaningful based on expert opinion and can have important implications in real‐world applications, potentially impacting decisions regarding conservation and management policies (EFSA Scientific Committee, 2011). The size of a biological effect (i.e., effect size) that would be considered relevant or meaningful should be defined a priori through methods such as power analyses (Martinez‐Abrain, 2008; Steidl et al., 1997; Taylor & Gerrodette, 1993). The issue of power has been addressed in other review papers in salmonids, particularly in regard to limitations in power in hatchery‐ versus natural‐origin LRS studies (Araki et al., 2008; Christie et al., 2014). However, across the studies reviewed here, only a limited number provided either a priori or retrospective power analyses to determine the minimum effect size that would be detectable under varying degrees of power (Araki, Ardren, et al., 2007; Araki, Cooper, et al., 2007; Berejikian et al., 2009; Hess et al., 2012; Mobley et al., 2020; Thériault et al., 2011).

Confidence intervals are often favored over power analyses to convey information on the range of effect sizes that are supported by the data (Colegrave & Ruxton, 2003; Lovell, 2013); yet again, these were not consistently presented across the studies reviewed here (e.g., Anderson et al., 2013; Christie et al., 2018; Janowitz‐Koch et al., 2019; Roth et al., 2019). Ideally, studies assessing fitness in salmonids would demonstrate both statistical and biological significance. However, other scenarios typically unfold, such as statistical significance without biological significance or biological significance without statistical significance. Both scenarios provide valuable information to researchers, each lending information regarding study limitations and directions for future research, such as sample sizes that are too small to detect effects or variability in traits that is too wide to be explained by a single variable of interest (Lovell, 2013; Martinez‐Abrain, 2008). Therefore, demonstrating power to detect effects is an extremely useful tool, particularly in conservation management scenarios where sample sizes tend to be small and accepting a false null hypothesis based on a single α cutoff value could have substantial consequences (Taylor & Gerrodette, 1993).

### 5.8 Limitations in pedigree reconstruction

The ability to sample all potential parents in any given year remains a challenge in multiple salmonid populations namely due to logistical and technical constraints, sometimes in conjunction with conservation limitations. The decreased ability to reconstruct pedigrees due to incomplete sampling may be particularly problematic for datasets with an insufficient number of genetic markers as investigated previously (Aykanat et al., 2014; Harrison et al., 2013). Additionally, Araki and Blouin (2005) demonstrated that an increase in incomplete sampling can increase the overall proportion of incorrectly assigned parents. Numerous parentage programs account for the proportion of candidate parents sampled in a study, with some parental reconstruction methods more sensitive to this variable compared to others (Jones et al., 2010). Similarly, relatedness between potential parents can affect parental assignment success, with a reduction in the overall assignment success of offspring whose parents are more related and less heterozygous overall (Ford & Williamson, 2010; Olsen et al., 2001). Therefore, to increase the validity of results in salmonid LRS studies, it remains particularly important to choose appropriate parental reconstruction methods, provide estimates of power limitations and potential sources of error, such as genotyping or measurement error, determine the appropriate thresholds for accepting assignments, and maximize genomic marker panels whenever possible (see e.g., Flanagan & Jones, 2019 which reviews next‐generation genotyping approaches in parentage analysis).

### 5.9 Applications for conservation management

Overall, estimating productivity through LRS‐based studies remains a vital component of protecting and maintaining declining salmonid populations. General declines in salmonids are namely thought to be the result of anthropogenic impacts, including but not limited to habitat loss, overharvest, unintended effects of hatcheries, and hydropower dams, each with potential to propel rapid evolutionary shifts (McClure et al., 2003; Stockwell et al., 2003; Waples & Hendry, 2008). In this review, we uncovered extensive variation in salmonid LRS and in the factors affecting LRS, with a heavy emphasis on factors related to hatchery rearing. We see, however, that salmonid life history adds further complexity to patterns in LRS and can interact with other variables to predict LRS. Density and environmental factors, for example, are innately related to anthropogenic impacts, and numerous studies presented in this review suggest that these factors may directly affect LRS or can interact with life history to shape patterns in LRS. Therefore, while the complexity in life‐history forms and variation in LRS make salmonids ideal study species to address evolutionary questions, this can further complicate conservation questions. As such, the inclusion of the vast array of life‐history strategies, environmental drivers, and both phenological and phenotypic traits in studies estimating salmonid LRS remains extremely important in synthesizing both species‐specific and population‐specific conservation decisions.

## 6 CONCLUSIONS

In summary, studies of LRS have provided critical insights toward understanding fitness advantages within salmonid populations and have expanded our general knowledge of salmonid mating behavior and reproductive strategies. Further, these studies have provided a basis for ongoing conservation and management decisions for salmonid species that have vital ecological, economic, and cultural roles throughout their geographic ranges. Future studies should aim to (1) continue to expand the breadth of studies assessing LRS across different life‐history strategies, specifically accounting for different reproductive and migratory phenotypes, (2) broaden the array of species represented in salmonid fitness studies, (3) construct multigenerational pedigrees to measure long‐term fitness effects, (4) expand on LRS studies that investigate the effects of largely untested traits, such as aquatic stressors including, but not limited to, environmental factors, pathogens, and general body condition, and (5) utilize appropriate statistical approaches to determine the factors that explain the greatest variation in fitness models and provide details on biological significance, power limitations, and potential sources of error whenever possible. These challenging studies have profoundly advanced scientific understanding that will continue to assist with long‐term perseverance of these keystone species in aquatic ecosystems.

## Data Availability

Data sharing is not applicable to this article as no new data were created or analyzed in this study.
